# Prevalence and correlates of imposter syndrome and self-esteem among medical students at Jazan University, Saudi Arabia: A cross-sectional study

**DOI:** 10.1371/journal.pone.0303445

**Published:** 2024-05-09

**Authors:** Maged El-Setouhy, Anwar M. Makeen, Ahmad Y. Alqassim, Riyadh A. Jahlan, Malik I. Hakami, Hussam T. Hakami, Ibrahim M. Mahzari, Hussam Aldeen A. Thubab, Khalid Y. Haroobi, Hassan A. Alaraj, Hazem M. El-Hariri

**Affiliations:** 1 Faculty of Medicine, Department of Family and Community medicine, Jazan University, Jazan, Kingdom of Saudi Arabia; 2 Faulty of Medicine, Department of Community, Environmental and Occupational Medicine, Ain Shams University, Cairo, Egypt; 3 Department of Community Medicine, National Research Centre, Cairo, Egypt; Jahangirnagar University, BANGLADESH

## Abstract

Imposter syndrome (IS) and low self-esteem (SE) are common issues affecting medical students that can impact their well-being and development. This study aimed to assess the prevalence and factors associated with IS and SE among medical students at Jazan University, Saudi Arabia. In this cross-sectional study, 523 medical students in years 2–6 at Jazan University, Saudi Arabia, completed validated questionnaires on IS (Young Imposter Scale) and SE (Rosenberg Self-Esteem Scale). Sociodemographic factors were also collected. Descriptive statistics and logistic regression analyses were used to analyze IS and SE prevalence and correlates. Five hundred twenty-three students with a mean age of 22.09 ± 1.933 participated. The prevalence of low SE and positive IS was 17.6% and 24.3%, respectively. IS and SE had a significant negative correlation (p<0.001). Several sociodemographic factors were associated with increased IS, including 2nd and 4th-year students, forced study choice, and a grade point average (GPA) of 3.0–3.49 (P<0.05). Paternal education beyond high school was associated with lower IS (P<0.05). Logistic regression analyses confirmed that 2nd-year students had a 3.88 times higher odds ratio (OR) (95% confidence interval (CI); 2.19–6.88), and 4th-year students had a 2.37 times higher OR (95% CI; 1.40–4.02) of IS than other years. For SE, advanced academic years, forced study choice, 7+ hours of sleep, and a GPA above 3.5 were associated with higher levels (P<0.05). Negative self-appraisals were associated with lower SE, while positive attitudes were associated with higher SE (P<0.05). Our study reveals that IS and low SE are prevalent among Jazan University, Saudi Arabia, medical students. Therefore, intervention courses that address these issues in medical education at Jazan University, Saudi Arabia, may be necessary to support medical students’ well-being and academic success.

## Introduction

Impostor Syndrome (IS) is a psychological entity that has recently gained popularity in the scientific literature, with an increased focus on medical professionals and high-performing individuals [1–5]. It is “a behavioral health phenomenon described as self-doubt of intellect, skills, or accomplishments among high-achieving individuals” [6]. Even with proof showing otherwise, individuals with IS struggled to recognize their achievements, abilities, or expertise. Consequently, they feel they are not as smart or capable as others [7]. As a result, they experience self-doubt and fear that others will discover them as fraudulent intellectuals [8].

High Self-Esteem (SE) is usually associated with an excellent mental state, self-harmony, competence, confidence, increased productivity, optimism, problem-solving, and emotional skills. In contrast, low SE can lead to desperation, inferiority, hopelessness, sadness, and depression. In medical school, SE is crucial [9,10]. That is why IS could affect a person’s self-esteem (SE), which is "a sense of validity, acceptance, approval, and worthiness" [11,12].

Many stressors affect medical students, including social, medical school requirements and their fight for high achievements [13–17]. Moreover, the transition to the clinical years can be particularly challenging and cause students to suffer from low SE and confidence during this time [18]. As a result, they believe themselves to be less competent and intelligent than what they believe to be the case with their colleagues, teachers, parents, and society at large [18]. Therefore, medical school is demanding, and medical students are subjected more to low Self Esteem and Imposter Syndrome [19].

According to a recent review in the United States, IS prevalence ranged from 9% to 82% in the general population. At the same time, another study created in the same year found that the prevalence of the syndrome ranges from 22% to 60% among physicians and from 33% to 40% among trainee physicians [20,21] Other studies showed that 57% of pharmacy and 15% of medical students have IS in the United States [22,23]. In addition, the prevalence of IS among medical students was 45.7% in Malaysia, 47.8% in Brazil, and 62.6% in Pakistan. Some studies indicated that IS frequently harms medical students’ physical and academic well-being [24–26].

In Saudi Arabia, IS and low SE were studied among medical students in central and northern regions. The prevalence of IS ranged from 23% to 57%. Two studies recorded a positive relationship between IS and low SE [2,27].

According to the available research, there have been few studies on IS and SE in Saudi Arabia. To our knowledge, no studies have been conducted to estimate their prevalence in the South of the Kingdom. Adding to this that Jazan region, Saudi Arabia, is a frontier region as well in adjacent to Yemen. Therefore, we aimed to determine the prevalence and determinates of IS in relation to different levels of SE among Jazan University, Saudi Arabia, medical students during the school year 2022–2023.

## Methods

### Study area design and population

This study is a quantitative analytical cross-sectional study, and it was conducted among medical students at the Jazan University, Saudi Arabia, campus between January 2022 and May 2022. We included all male and female Jazan University, Saudi Arabia, medical students during their 2^nd^, 3^rd^, 4^th^, 5^th,^ and 6^th^ years of medical school who registered for the academic year 2022/2023. First year was not included as it is a general year and not a medical school year. Students of the first year also sties in a different campus with all other university students. we did not put specific inclusion criteria to include all medical students.

### Sampling procedures

Bases on PASS 11th release 2011 [28] the minimum sample size required was 352 produces a two-sided 95% confidence interval with a width equal to 0.010 when the sample proportion is 0.320 [23]. However, with the different none response rates reported in Saudi studies used self-administered questionnaire [29–31], we decided to invite all registered medical students for the academic year 2022–2023 to participate in this study through our self-administered questionnaire. Data were collected by the investigators at the campus of the Faculty of Medicine, Jazan University, Saudi Arabia, using an anonymous, self-administered, assisted questionnaire. Our group delivered the questionnaire to all medical students, including informed consent during their break time in the classroom for each academic year, starting from the second year to the sixth year for both male and female sections.

### Data collection tool

Data were collected using an anonymous questionnaire consisting of 38 questions. The questionnaire was divided into three sections. The first section collected sociodemographic data, including age, gender, medical year, academic performance by grade point average (GPA), family monthly income, weight, height, daily sleep hours, and the reasons for studying medicine. The second section assessed the prevalence of SE using the Rosenberg Self-Esteem Scale (RSE) [8,32], a 10-item questionnaire with a 4-point Likert scale (0 = strongly agree, 1 = agree, 2 = disagree, 3 = strongly disagree). The total score ranges from 0 to 30, with higher scores indicating a higher SE. A cut-off value of <16 was used to define an individual as having low SE [33].

The third section of the questionnaire consisted of the eight items of the Young Imposter Syndrome (YIS) scale to assess whether the participant had IS or not. A student was considered to have IS if they answered "yes" to five or more questions. The YIS and the RSES were adapted without alteration from previously published research studies and are considered reliable [27,34].

### Operational definitions

***GPA*:** Grade Point Average is considered as 95–100% = 5, 90% to less than 95% = 4.75, 85% to less than 90% = 4.5, 80% to less than 85% = 4, 75% to less than 80% = 3.5, 70% to less than 75% = 3, 65% to less than 70% = 2.5, 60% to less than 65% = 2, Less than 60% = 1 [35].

***BMI*:** Body Mass Index = weight in kilograms divided by height in meters squared. It is then expressed in five categories. Below 18.5 = Underweight, 18.5 to 24.9 = Normal (Healthy) weight, 25.0 to 29.9 = Overweight, 30 or higher = Obese, 40 or higher = Extremely obese (Class 3 Obesity) [36].

***SE*: *Self-Esteem*:** One’s positive or negative attitude toward oneself and one’s evaluation of one’s own thoughts and feelings overall in relation to oneself. Someone who regards themselves positively has high self-esteem, while those who view themselves negatively have low self-esteem [32].

### Data analysis

The data were analyzed using SPSS software version 27. Descriptive statistics (mean, standard deviation, frequencies, and percentages) were used to describe the quantitative and categorical variables. Bivariate statistical analysis was carried out using appropriate (chi-square and logistic regression) statistical tests based on the type of study and outcome variables. A P <0.05 and 95% confidence interval (CI) were used to report the statistical significance and precision of the results.

### Ethical considerations

The Jazan University, Saudi Arabia, Scientific Research Ethics Committee ethically approved the study with reference number REC-44/06/459. Informed consent forms were read, understood, and verbally accepted by students who agreed to participate in the study. All students were informed of their right to not participate or withdraw from the study at any time. The confidentiality and privacy of the data were ensured.

## Results

Five hundred and twenty-three students participated in this study with a mean age of 22.09 ± 1.9. Table 1 shows the sociodemographic characteristics of the participants. In 58.7% of the participants were males. More than one third of them had monthly family income >20,000 Saudi Riyal (SR). Most of them had educated parents with at least a bachelor’s degree. Nearly half of the respondents had a GPA of >4.5 and normal BMI.

**Table 1:** Sociodemographic characteristics of Jazan University, Saudi Arabia, medical students (n = 523).

Variable	Number (%) / Mean±SD
**Age, mean (±Standard Deviation))**	22.1±1.9
**Gender**	
Male	307 (58.7)
Female	216 (41.3)
**Medical School Year**	
2^nd^ year	93 (17.8)
3^rd^ year	112 (21.4)
4^th^ year	111 (21.2)
5^th^ year	80 (15.3)
6^th^ year	127 (24.3)
**Monthly Family Income (SR** * **)**	
<5000	45 (8.6)
5000–1000	95 (18.2)
10000–20000	179 (34.2)
>20000	204 (39.0)
**Father’s Educational Level**	
Illiterate	17 (3.3)
Elementary school	51 (9.8)
High school	79 (15.1)
Diploma	65 (12.4)
Bachelor’s degree	266 (50.8)
Postgraduate degree	45 (8.6)
**Mother’s Educational Level**	
Illiterate	48 (9.2)
Elementary school	81 (15.5)
High school	93 (17.7)
Diploma	69 (13.2)
Bachelor’s degree	221 (42.3)
Postgraduate degree	11 (2.1)
**GPA**	
<3	20 (3.8)
3.00–3.49	39 (7.5)
3.50–3.99	73 (14.0)
4–4.49	134 (25.6)
>4.5	257 (49.1)
**Body Mass Index (BMI)**	
Underweight	87 (16.6)
Normal weight	260 (49.8)
Overweight	102 (19.5)
Obese	44 (8.4)
Extremely obese	30 (5.7)

*SR: Saudi Riyal (U$1 = SR 3.75).

Most respondents exhibited signs of IS, particularly those related to perfectionism and self-doubt. However, fewer respondents expressed extreme fears of inadequacy or being revealed as a fraud. The results showed that most medical students, 75.7% (396), had negative IS. On the other hand, our results showed that low, normal, and high SE prevalence was 17.6%, 64.6%, and 17.8% (92, 338, and 93 students), respectively.

In comparing positive IS in grades of SE, positive IS was most frequent in low SE (66.3%), followed markedly by normal SE (18.6%), and least frequent in high SE (3.2%), the differences between all SE grades were significant (<p-value <0.001) (Table 2).

**Table 2:** The relation between imposter syndrome and self-esteem among medical students at Jazan University, Saudi Arabia (*n* = 523).

**Self-esteem type**	**Imposter syndrome type**		**p-value**
	**Negative**	**Positive**	<0.001*
Low	31 (33.7%)	61 (66.3%)a	
Normal	275 (81.4%)	63 (18.6%)b	
High	90 (96.8%)	3 (3.2%)c	

Data presented as n (%). Percentages were taken from total of rows.

Chi square test with post hoc Bonferroni test, homogenous subgroups had the same symbol.

P < 0.05 (Statistically Significant*).

Table 3 shows the associations between sociodemographic characteristics and IS. We found a statistical association between IS and academic year (P-value = 0.001), the reason for choosing to study medicine (P-value = 0.004), and GPA (P-value = 0.023). IS was not associated with gender, hours of sleep, monthly family income, father educational level, mother educational level, nor the BMI.

**Table 3:** Association of categorical study variables with imposter syndrome (*n* = 523).

Variable	Negative imposter Syndrome N (%)	Positive imposter Syndrome N (%)	P-value
**Gender**			
Male	227 (73.9)	80 (26.1)	0.259
Female	169 (78.2)	47 (21.8)	
**Academic Year**			
2^nd^ year	58 (62.4)	35 (37.6)	0.001*
3^rd^ year	93 (83.0)	19 (17.0)	
4^th^ year	77 (69.4)	34 (30.6)	
5^th^ year	66 (82.5)	14 (17.5)	
6^th^ year	102 (80.3)	25 (19.7)	
**Reason for Choosing to study Medicine**			
Own preference	361 (77.6)	104 (22.4)	0.004*
Family preference	35 (60.3)	23 (39.7)	
**Hours of Sleep**			
<4	29 (65.9)	15(34.1)	0.275
4–8	283 (76.9)	85 (23.1)	
>8	84 (75.7)	27 (24.3)	
**Monthly Family Income (SR)**			
<5000	31 (68.9)	14 (31.1)	0.634
5000–1000	70 (73.7)	25 (26.3)	
10000–20000	138 (77.1)	41 (22.9)	
>20000	157 (77.0)	47 (23.0)	
**Father’s Educational Level**			
Unable to read and write	9 (52.9)	8 (47.1)	0.128
Elementary school	34 (66.7)	17 (33.3)	
High school	63 (79.7)	16 (20.3)	
Diploma	52 (80.0)	13 (20.0)	
Bachelor’s degree	204 (76.7)	62 (23.3)	
Postgraduate degree	34 (75.6)	11 (24.4)	
**Mother’s Educational Level**			
Unable to read and write	37 (77.1)	11 (22.9)	0.328
Elementary school	58 (71.6)	23 (28.4)	
High school	68 (73.1)	25 (26.9)	
Diploma	51 (73.9)	18 (26.1)	
Bachelor’s degree	176 (79.6)	45 (20.4)	
Postgraduate degree	6 (54.5)	5 (45.5)	
**GPA**			
<3	10 (50.0)	10 (50.0)	0.023*
3.00–3.49	25 (64.1)	14 (35.9)	
3.50–3.99	58 (79.5)	15 (20.5)	
4–4.49	105 (78.4)	29 (21.6)	
>4.5	198 (77.0)	59 (23.0)	
**BMI**			
Underweight	69 (79.3)	18 (20.7)	0.318
Normal	200 (76.9)	60 (23.1)	
Overweigh	78 (76.5)	24 (23.5)	
Obese	30 (68.2)	14 (31.8)	
Extremely obese	19 (63.3)	11 (36.7)	

N: Number. (%): Percentage. Percentages were taken from total of rows.

P < 0.05 (Statistically Significant*).

SR: Saudi riyal. GPA: Grade point average. BMI: Body mass index.

The relation between the categorical study variables and the levels of SE is displayed in Table 4. The variables of academic year, the reason for choosing to study medicine, hours of sleep, and GPA were significantly associated with SE. On the other hand, gender, monthly family income, father’s educational level, mother’s educational level, and BMI were not significantly associated with SE characteristics.

**Table 4:** Association of categorical study variables with levels of self-esteem (*n* = 523).

Variable	Low Self-Esteem, N (%)	Normal Self-Esteem, N (%)	High Self-Esteem, N (%)	P-value
**Gender**				
Male	52 (16.9)	199 (64.1)	56 (18.2)	0.873
Female	40 (18.5)	139 (64.4)	37 (17.1)	
**Medical School Year**				
2^nd^ year	28 (30.1)	54 (58.1)	11 (11.8)	0.004*
3^rd^ year	16 (14.3)	66 (58.9)	30 (26.8)	
4^th^ year	21 (18.9)	69 (62.2)	21 (18.9)	
5^th^ year	9 (11.2)	59 (73.8)	12 (15.0)	
6^th^ year	18 (14.1)	90 (70.9)	19 (15.0)	
**Reason for Study Choice**				
Own preference	78 (16.8)	297 (63.9)	90 (19.4)	0.020*
Family preference	14 (24.1)	41 (70.7)	3 (5.2)	
**Hours of Sleep**				
<4	12 (27.3)	26 (59.1)	6 (13.6)	0.020*
4–8	55 (14.9)	237 (64.4)	76 (20.7)	
>8	25 (22.5)	75 (67.6)	11 (9.9)	
**Monthly Family Income (SR)**				
<5000	10 (22.2)	31 (68.9)	4 (8.9)	0.593
5000–1000	19 (20.0)	60 (63.2)	16 (16.8)	
10000–20000	26 (14.5)	119 (66.5)	34 (19.0)	
>20000	37 (18.1)	128 (62.8)	39 (19.1)	
**Father’s Educational Level**				
Illiterate	5 (29.4)	12 (70.6)	0 (0.0)	0.398
Elementary school	13 (25.5)	29 (56.9)	9 (17.6)	
High school	12 (15.2)	50 (63.3)	17 (21.5)	
Diploma	10 (15.3)	43 (66.2)	12 (18.5)	
Bachelor’s degree	41 (15.4)	176 (66.2)	49 (18.4)	
Postgraduate degree	11 (24.4)	28 (66.3)	6 (13.3)	
**Mother’s Educational Level**				
Illiterate	6 (12.5)	35 (72.9)	7 (14.6)	0.490
Elementary school	17 (21.0)	51 (63.0)	13 (16.0)	
High school	15 (16.1)	61 (65.6)	17 (18.3)	
Diploma	12 (17.4)	43 (62.3)	14 (20.3)	
Bachelor’s degree	38 (17.2)	145 (65.6)	38 (17.2)	
Postgraduate degree	4 (36.4)	3 (27.2)	4 (36.4)	
**GPA**				
*<*3	8 (40.0)	12 (60.0)	0 (0.0)	0.001*
3.00–3.49	11 (28.2)	24 (61.5)	4 (10.3)	
3.50–3.99	9 (12.2)	59 (80.8)	5 (6.8)	
4–4.49	26 (19.4)	82 (61.2)	26 (19.4)	
>4.5	38 (14.8)	161 (62.6)	58 (22.6)	
**BMI**				
Underweight	13 (14.9)	52 (59.8)	22 (25.3)	0.119
Normal	53 (20.4)	159 (61.2)	48 (18.4)	
Overweigh	14 (13.7)	72 (70.6)	16 (15.7)	
Obese	9 (20.5)	30 (68.2)	5 (11.3)	
Extremely obese	3 (10.0)	25 (83.3)	2 (6.7)	

N: Number. (%): Percentage. Percentages were taken from total of rows.

P < 0.05 (Statistically Significant*).

SR: Saudi riyal. GPA: Grade point average. BMI: Body mass index.

The first part of Table 5 (imposter syndrome section) presents the logistic regression results of positive IS related demographic and academic independent factors. Regarding the academic year, students in their 2nd year have a significantly higher likelihood of experiencing IS with an odds ratio (OR) of 3.88 (95% CI: 2.19–6.88) and a coefficient (β) of 1.36 (SE = 0.29, p<0.001). Similarly, 4th-year students exhibit a heightened risk, though to a lesser extent, with an OR of 2.37 (95% CI: 1.40–4.02) and a β value of 0.86 (SE = 0.27, p = 0.001). Interestingly, the reason behind a student’s choice of study also plays a role. Those forced into their field of study have an OR of 2.23 (95% CI: 1.19–4.17) of experiencing IS, with a β value of 0.80 (SE = 0.32, p = 0.012).

**Table 5:** Logistic regression of demographic and other factors related to imposter syndrome and low self-esteem.

Factors	β	SE	P-value	Odds Ratio (CI 95%)
**Factors related to imposter syndrome (positive versus negative IS)**				
Academic Year (2nd year)	1.36	0.29	<0.001	3.88 (2.19–6.88)
Academic Year (4th year)	0.86	0.27	0.001	2.37 (1.40–4.02)
Reason for study choice (Forced)	0.80	0.32	0.012	2.23 (1.19–4.17)
Father’s education level (from elementary to postgraduate). (Illiterate was a reference)			<0.001	
Elementary school	-1.25	0.35	<0.001	0.29 (0.14–0.56)
High school	-2.26	0.35	<0.001	0.10 (0.05–0.21)
Diploma	-1.95	0.37	<0.001	0.14 (0.07–0.30)
Bachelor’s degree	-2.02	0.25	<0.001	0.13 (0.08–0.22)
Postgraduate degree	-2.11	0.43	<0.001	0.12 (0.05–0.28)
GPA (from 3.00 to 4.49) (<3 was a reference)			0.017	
GPA 3.00–3.49	1.44	0.50	0.004	4.24 (1.58–11.37)
GPA 3.50–3.99	0.91	0.40	0.022	2.49 (1.14–5.47)
GPA 4–4.49	0.15	0.35	0.668	1.16 (0.58–2.33)
GPA >4.5	0.09	0.27	0.740	1.09 (0.64–1.86)
**Factors related to low self-esteem**				
Academic Year (2nd year)	0.84	0.31	0.007	2.31 (1.25–4.27)
Academic Year (4th year)	0.92	0.30	0.002	2.51 (1.38–4.54)
Reason for study choice (forced)	0.93	0.36	0.009	2.55 (1.27–5.11)
Sometimes, I feel like I’m not worth much. (Strongly Agree was a reference)			<0.001	
Sometimes, I feel like I’m not worth much. (Agree)	-0.63	0.39	0.107	0.53 (0.25–1.15)
Sometimes, I feel like I’m not worth much. (Disagree)	-1.73	0.43	<0.001	0.18 (0.08–0.41)
Sometimes, I feel like I’m not worth much. (Strongly Disagree)	-2.22	0.56	<0.001	0.11 (0.04–0.32)
Overall, I tend to believe that I have failed. (Strongly Agree was a reference)			<0.001	
Overall, I tend to believe that I have failed. (Agree)	-0.39	0.46	0.397	0.68 (0.28–1.66)
Overall, I tend to believe that I have failed. (Disagree)	-1.05	0.45	0.019	0.35 (0.14–0.84)
Overall, I tend to believe that I have failed. (Strongly Disagree)	-1.94	0.45	<0.001	0.14 (0.06–0.35)
I have a positive view of myself. (Strongly Agree was a reference)			<0.001	
I have a positive view of myself. (Agree)	0.53	0.32	0.095	1.71 (0.91–3.20)
I have a positive view of myself. (Disagree)	1.47	0.36	<0.001	4.34 (2.13–8.84)
I have a positive view of myself. (Strongly Disagree)	1.65	0.59	0.005	5.23 (1.65–16.52)

Abbreviations: β: Regression coefficient. SE: Standard error. Statistically significant: P <0.05.

CI = Confidence Interval. GPA: Grade point average.

With "illiterate" as the reference category, the father’s education level significantly correlates with IS. Students with fathers who completed elementary or high school and obtained a diploma, bachelor’s degree, or postgraduate degree are less likely to experience IS. The respective OR and 95% confidence intervals for these education levels are 0.29 (0.14–0.56), 0.10 (0.05–0.21), 0.14 (0.07–0.30), 0.13 (0.08–0.22), and 0.12 (0.05–0.28), with all p-values being less than 0.001. Lastly, there are varied associations concerning GPA (with a reference to GPA <3). Students with GPAs in the range of 3.00–3.49 have a notably higher likelihood of IS with an OR of 4.24 (95% CI: 1.58–11.37). Those with GPAs between 3.50 and 3.99 also have an elevated risk, with an OR of 2.49 (95% CI: 1.14–5.47). However, students with a GPA of 4–4.49 and greater than 4.5 do not show a significant association, with ORs of 1.16 and 1.09, respectively.

The second part of Table 5 (low self-esteem section) elucidates the logistic regression analysis results about academic, attitudinal, and self-perception factors associated with low SE. From an academic perspective, students in their 2nd year demonstrate a higher propensity for SE, with a β coefficient of 0.84 (SE = 0.31, p = 0.007) and an OR of 2.31 (95% CI: 1.25–4.27). The 4th-year students exhibit a similar trend, albeit slightly elevated, with a β value of 0.92 and an OR of 2.51 (95% CI: 1.38–4.54). Students forced into their field of study were 2.55 times more likely to have higher SE (95% CI: 1.27–5.11).

Attitudinal factors play a pivotal role in shaping SE. For the statement "At times I think I am not good at all," using "strongly agree" as the reference, those who "agree" have an OR of 0.53 (95% CI: 0.25–1.15), while those who "disagree" and "strongly disagree" have substantially lower ORs of 0.18 (95% CI: 0.08–0.41) and 0.11 (95% CI: 0.04–0.32), respectively. Regarding the sentiment "All in all, I am inclined to feel that I am a failure," with "strongly agree" as the reference, students who "agree" have an OR of 0.68 (95% CI: 0.28–1.66). However, those who "disagree" and "strongly disagree" indicate a markedly reduced likelihood of feeling like a failure, with OR = 0.35 (95% CI: 0.14–0.84) and 0.14 (95% CI: 0.06–0.35), respectively.

Lastly, in response to the statement "I take a positive attitude toward myself," using "strongly agree" as the benchmark, students who "agree" display an increased OR of 1.71 (95% CI: 0.91–3.20). Conversely, those who "disagree" and "strongly disagree" manifest significantly heightened OR = 4.34 (95% CI: 2.13–8.84) and 5.23 (95% CI: 1.65–16.52), signifying stronger self-affirming sentiments.

## Discussion

Imposter Syndrome is typified by a persistent sense of self-doubt and a fear of being exposed as a fraud despite clear evidence of success. This syndrome is often observed in high achievers and can harm their mental health and well-being [3,37]. We aimed in this study to investigate the prevalence of IS and SE among medical students at Jazan University, Saudi Arabia, compare their prevalence across genders, academic years, and examine their relationship with sociodemographic characteristics. This study revealed a high prevalence rate of IS (24.3%) among medical students, while a majority (64.6%) displayed normal SE levels. Thus, many students experienced IS without it affecting their self-perception. This divergence from previous studies reporting IS prevalence ranging from 9% to 82% can be attributed to differing assessment tools and thresholds [20]. Comparison with past studies showed a range of results. One study found that 45.2% of medical students experienced IS, with 18.6% having low SE [38]. Another reported 93.2% of surgical and medical residents in Makkah, Saudi Arabia, showed moderate to high imposter characteristics [39]. On the other hand, in Riyadh, the capital of Saudi Arabia, the prevalence of IS was shown to be 42.1% among King Saud University medical students [18]. While a recent Indian study found that 56.7% and 16.11% of students in medical colleges had IS and high SE, respectively [40]. The varied prevalence across different populations underscores IS commonality among high achievers like medical students [41].

The current study’s correlation between IS and SE aligns with substantial prior research [20,27,38]. Lower SE often coincided with pronounced IS experiences, reinforcing that SE is a robust predictor of IS [20,38,40,42]. Therefore, addressing SE issues may help alleviate IS’s impact on individuals’ mental health and well-being. The study also found significant associations between IS and SE with factors like academic year, reasons for choosing medicine, and GPA, indicating these factors could influence IS’s emergence or exacerbation. The link between IS and the academic year may reflect the increasing academic demands of students’ progress, echoing previous findings [20,38,42]. Factors such as education level, female sex, and academic achievements have been identified as IS predictors [43]. IS can lead to increased anxiety, depression, and burnout rates [37,44], and it may develop in diverse and complex environments, leading to feelings of isolation and negative self-evaluation, especially among women and underrepresented minorities [38]. Some institutions have implemented wellness programs to help individuals cope with IS. However, to manage IS, people must appreciate their successes, establish a positive self-perception, obtain peer or mentor support, and engage in health activities [37,45].

IS was more prevalent among earlier-year students, those with lower GPA, and those who did not consciously choose medicine. Lower SE was correlated with similar factors: earlier academic years, lower GPAs, less sleep, and not deliberately selecting medicine as a career. The results suggest that IS and weaker self-confidence may be more common among students new to clinical medical training (2th academic year in Saudi Arabia). Providing additional support during the early academic years could potentially mitigate these psychological experiences and promote student well-being. However, no significant correlation was found between IS and SE with gender, family income, parents’ educational level, or BMI, contradicting some previous studies [46–48], as while some research suggests women experience higher IS rates, others found no gender disparity [27,41,46,49,50].

Our logistic regression analysis provided important insights into factors associated with IS and SE among medical students. Regarding IS, students in their 2nd and 4th years showed significantly high ORs, with ORs of 3.88 and 2.37, respectively. This aligns with existing literature, which foud imposter fears tend to increase over time in medical education as pressures mount [51]. Providing targeted support and mentorship as students’ advance through demanding curricula could help mitigate escalating imposter feelings. Additionally, students reporting forced study choices had a 2.23 times higher OR than IS. This highlights the need for motivational interviewing during the career decision process to ensure students feel intrinsically driven rather than pressured into medicine [19]. Fostering autonomous motivation may buffer against future imposter phenomena. Higher paternal education was associated with lower IS, suggesting parental modeling shapes the development of imposter fears. The association between family environment and IS warrants further investigation. Regarding SE, negative self-appraisals were strongly predictive of lower SE, while positive attitudes correlated with higher SE. This reiterates the need to combat cognitive distortions and promote self-affirming beliefs through cognitive behavioral techniques [52–54].

These results emphasize the need for further research into the factors contributing to SE and IS among medical students at Jazan University, Saudi Arabia. Future studies should aim to replicate these findings in other academic settings and consider a longitudinal design to explore these factors’ evolution over time. The significant IS prevalence, its association with various sociodemographic characteristics, and most students maintaining normal SE levels provide fertile ground for further investigation into medical students’ psychological well-being.

### Study limitations

This study offers a valuable snapshot of the prevalence of IS and SE among medical students at Jazan University, Saudi Arabia. However, its cross-sectional design prevents us from observing changes or establishing causal relationships over time. The geographical scope of the study, solely conducted at Jazan University, Saudi Arabia, may also limit the generalizability of the findings to other regions or countries. A notable limitation is the reliance on self-reported data, which introduces the potential for bias. Participants may, consciously or not, over- or under-estimate their feelings of SE or IS due to social desirability bias or a lack of introspection. While the study diligently assesses several sociodemographic variables, other confounding variables could remain unmeasured. These could include personal history, mental health status, or cultural background, which could influence SE and IS.

### Study strengths

Although of the limitations inviting all medical students to participate in the study was a point of strength. Moreover, our regression analysis added to the strength of our results.

## Conclusions

Our study unearthed compelling insights into the prevalence of SE and IS among medical students. Many students demonstrated normal SE, while a sizable group reported negative IS. Interestingly, we discovered a significant association between IS and SE, with certain factors playing a pivotal role. These include the year of study, the reasons for choosing to study medicine, and academic performance. On the other hand, aspects such as gender, family income, parents’ educational background, and physical health indicators like BMI didn’t show a substantial correlation. Feelings of fraudulence and self-doubt were pronounced, particularly among students in more advanced academic years. Negative self-appraisals were associated with lower SE, while positive attitudes correlated with higher SE. These findings highlight the need for targeted interventions to support medical students’ well-being and resilience. Promoting self-compassion, cognitive reappraisal of perceived failures, and growth mindsets may help protect against IS. Additionally, universities should provide adequate counseling services and destigmatize seeking mental health support. Addressing the high rates of IS and low SE has important implications for improving medical students’ psychological outcomes, professional development, and the quality of care they provide in future practice.

## Supporting information

S1 File (PDF)
