# Supplementary material for: Prevalence and correlates of imposter syndrome and self-esteem among medical students at Jazan University, Saudi Arabia: A cross-sectional study
Source: PLoS One. 2024 May 9;19(5):e0303445. doi: 10.1371/journal.pone.0303445 (PMC11081292; doi:10.1371/journal.pone.0303445)
Supplement: S1 File — (PDF) [file pone.0303445.s001.pdf]

The Prevalence of Imposter Syndrome, Low Self-Esteem, and their Determinants among Medical Students at Jazan University. A cross-sectional study.

نحن مجموعة بحثية من طلاب الطب بجامعة جازان تحت إشراف البروفيسور ماجد السطوحي والدكتور أنور مكين. كطالب طب في جامعة جازان، نطلب منك أن تكون جزءًا من دراستنا التي تهدف إلى تقدير مدى انتشار متلازمة الاحتيال واحترام الذات ومحدداتها بين طلاب الطب بجامعة جازان. مشاركتك في هذه الدراسة طوعية ويمكنك الانسحاب في أي وقت كما يمكنك رفض الإجابة على أي سؤال تعتقد أنه ليس مناسبًا بالنسبة لك. إذا قبلت المشاركة في هذه الدراسة. نطلب منك ملء الاستبيان المرفق. سيستغرق الاستبيان منك حوالي ١٠ دقائق. لن نطلب منك إضافة اسمك في الاستبيان، وستكون بياناتك سرية تمامًا، ولن يصل أي شخص إلى بياناتك باستثناء مجموعة البحث ولن يتم استخدام بياناتك أبدًا لأي سبب بخلاف مقترح الدراسة هذا. إذا تبين أن انتشار متلازمة الاحتيال مرتفعة بين طلاب الطب، فسوف نتواصل مع إدارة الكلية لتسهّل لنا إجراء دراسة تداخلية لتقليل أعراض وانتشار متلازمة وسيستفيد الطلاب من خلال تدخل آخر نخطط للقيام به في العام المقبل، سنعلن لمن يرغبون في المشاركة ويمكنهم المشاركة لاحقًا. هذه الدراسة ليس لها ضرر ولن تتأثر بملء هذا الاستبيان. سيتم نشر هذه الدراسة في مجلة جيدة لذا ستكون متاحة لك في أي وقت كما يمكنك معرفة النتيجة.

ملاحظة: من فضلك إذا كان لديك أي استفسار يخص الاستبيان والبحث، يمكنك التواصل مع:

-البروفيسور ماجد السطوحي: ٠٥٦٨٠٦٩٥١٥

-رياض جحلان: ٠٥٩٨٩٢٦٦٨١

إذا كنت مهتما بالمشاركة في هذه الدراسة، فيرجى الإشارة إلى اتفاقية موافقتك على المشاركة

○ (نعم, أوافق)

○ (لا, لا أوافق)

Personal Information

1. Age (العمر)

____

2. Medical year (السنة الأكاديمية)

1- ◯ **2nd year**

2- ◯ **3rd year**

3- ◯ **4th year**

4- ◯ **5th year**

5- ◯ **6th year**

3. Sex (الجنس)

1- ◯ **Male**

2- ◯ **Female**

4. What is your weight? (كم وزنك؟)

____

5. What is your height? *(كم طولك؟)

____

6. Reason for study choice. (سبب اختيارك الدراسة)

1- ◯ **Own preference** (رغبة شخصية)

2- ◯ **Family preference** (رغبة العائلة)

7. How many hours a day do you sleep? (كم ساعة تنام في اليوم؟)

1- ◯ **Less than 4 hours** (أقل من ٤ ساعات)

2- ◯ **4 -8 hours** (٤-٨ ساعات)

3- ◯ **More than 8 hours** (اكثر من ٨ ساعات)

8. Monthly family income (in SR) (دخل الأسرة الشهري)

1- ◯ <5000

2- ◯ 5000-10000

3- ◯ 10000-20000

4- ◯ >20000

9. Father's educational level (المستوى التعليمي للأب)

1- ◯ **Illiterate** (لا يقرأ ولا يكتب)

2- ◯ **Elementary School** (التعليم الابتدائي)

3- ◯ **High School** (التعليم الثانوي)

4- ◯ **Diploma** (الدبلوم)

5- ◯ **Bachelor's Degree** (جامعي - بكالوريوس)

6- ◯ **Post Graduate Degree** (دراسات عليا)

7- ◯ **Other** (أخرى) : _______________

10. Mother's educational level (المستوى التعليمي للأم)

1- ◯ **Illiterate (لا يقرأ ولا يكتب)**

2- ◯ **Elementary School (التعليم الابتدائي)**

3- ◯ **High School (التعليم الثانوي)**

4- ◯ **Diploma (الدبلوم)**

5- ◯ **Bachelor's Degree جامعي – بكالوريوس))**

6- ◯ **Post Graduate Degree (دراسات عليا)**

7- ◯ **Other (أخرى) : _______________**

Academic Information

11. GPA (المعدل الجامعي)

1- ◯ **<3.00**

2- ◯ **3.00 - 3.49**

3- ◯ **3.50 - 3.99**

4- ◯ **4.00 - 4.49**

5- ◯ **>4.5**

12. How often do you feel disappointed when your grades come out? (كم مرة تشعر بخيبة أمل عندما تظهر درجاتك؟)

	1	2	3	4	5	
(مطلقا) **Never**	◯	◯	◯	◯	◯	**Always** (دائما)

13. How satisfied are you towards your academic performance? (ما مدى رضاك عن أدائك الأكاديمي)

	1	2	3	4	
(غير راضٍ على الإطلاق) Not satisfied at all	◯	◯	◯	◯	Very satisfied (راضٍ جداً)

14. Do you think that the effort you give in your studies equals the gained outcome you get?

(هل تعتقد أن الجهد الذي تبذله في دراستك يساوي النتيجة المكتسبة التي تحصل عليها؟)

1- ◯ **Yes (نعم)**

2- ◯ **No (لا)**

3- ◯ **Sometimes (أحيانا)**

<u>ROSENBERG SELF-ESTEEM SCALE:</u>

Below is a list of statements dealing with your general feelings about yourself. Please indicate how strongly you agree or disagree with each statement.

فيما يلي قائمة بالعبارات التي تتعامل مع مشاعرك العامة عن نفسك. يرجى توضيح مدى قوة موافقتك أو عدم موافقتك على كل عبارة

15. On the whole, I am satisfied with myself. (بشكلٍ عام ، أنا راضٍ عن نفسي)

1- ◯ **Strongly Agree (اتفق بشدة)**

2- ◯ **Agree (اتفق)**

3- ◯ **Disagree (لا اتفق)**

4- ◯ **Strongly Disagree (لا اتفق بشدة)**

16. At times I think I am no good at all. (في بعض الأحيان أعتقد أنني لست جيدا على الإطلاق)

1- Strongly Agree (اتفق بشدة)

2- Agree (اتفق)

3- Disagree (لا اتفق)

4- Strongly Disagree (لا اتفق بشدة)

17. I feel that I have a number of good qualities. (أشعر أن لدي عددا من الصفات الجيدة)

1- Strongly Agree (اتفق بشدة)

2- Agree (اتفق)

3- Disagree (لا اتفق)

4- Strongly Disagree (لا اتفق بشدة)

18. I am able to do things as well as most other people. (أنا قادر على فعل الأشياء بشكل جيد مثل معظم الناس الأخرين)

1- Strongly Agree (اتفق بشدة)

2- Agree (اتفق)

3- Disagree (لا اتفق)

4- Strongly Disagree (لا اتفق بشدة)

19. I feel I do not have much to be proud of (أشعر أنه ليس لدي الكثير لأفخر به)

1- Strongly Agree (اتفق بشدة)

2- Agree (اتفق)

3- Disagree (لا اتفق)

4- Strongly Disagree (لا اتفق بشدة)

20. I certainly feel useless at times. (أشعر بأنني عديم الفائدة في بعض الأحيان)

1- Strongly Agree (اتفق بشدة)

2- Agree (اتفق)

3- Disagree (لا اتفق)

4- Strongly Disagree (لا اتفق بشدة)

21. I feel that I'm a person of worth, at least on an equal plane with others. (اشعر بأنني شخص ذو قيمة ، على الأقل على قدم المساواة مع الأخرين)

1- Strongly Agree (اتفق بشدة)

2- Agree (اتفق)

3- Disagree (لا اتفق)

4- Strongly Disagree (لا اتفق بشدة)

22. I wish I could have more respect for myself. (أتمنى أن احظى بمزيد من الاحترام لنفسي)

1- Strongly Agree (اتفق بشدة)

2- Agree (اتفق)

3- Disagree (لا اتفق)

4- Strongly Disagree (لا اتفق بشدة)

23. All in all, I am inclined to feel that I am a failure. (بشكل عام، أميل الى الشعور بأنني فاشل)

1- Strongly Agree (اتفق بشدة)

2- ◯ **Agree (اتفق)**

3- ◯ **Disagree (لا اتفق)**

4- ◯ **Strongly Disagree (لا اتفق بشدة)**

24. I take a positive attitude toward myself. (أتخذ موقفا إيجابيا تجاه نفسي **)**

1- ◯ **Strongly Agree (اتفق بشدة)**

2- ◯ **Agree (اتفق)**

3- ◯ **Disagree (لا اتفق)**

4- ◯ **Strongly Disagree (لا اتفق بشدة)**

Young Impostor Syndrome Scale

Below is a list of statements dealing with your general feelings about yourself. Please indicate how strongly you agree or disagree with each statement.

فيما يلي قائمة بالعبارات التي تتعامل مع مشاعرك العامة عن نفسك. يرجى توضيح مدى قوة موافقتك أو عدم موافقتك على كل عبارة

25. Do you secretly worry that others will find out that you're not a bright and capable as they think you are?

(هل تقلق من أن يكتشف الآخرون أنك لست ذكيًا وقادراً كما يعتقدون؟)

1- ◯ **Yes (نعم)**

2- ◯ **No (لا)**

26. Do you sometimes shy away from challenges because of nagging self-doubt?

(هل تخجل أحياناً وتنسحب من التحديات بسبب الانزعاج من عدم الثقة بالنفس؟)

1- ◯ **Yes (نعم)**

2- ◯ **No (لا)**

27. Do you tend to chalk your accomplishments up to being a "fluke", "no big deal" or the fact that people just "like" you? (fluke: an unexpected stroke of good luck).

(هل تميل إلى اعتبار إنجازاتك على انها "ضربة حظ"، أو "ليست مهمه" أو حقيقة أن الناس "يحبونك"؟ (حظ: ضربة حظ غير متوقعة)

1- ◯ **Yes (نعم)**

2- ◯ **No (لا)**

28. Do you hate making a mistake, being less than fully prepared, or not doing things perfectly?

هل تكره ارتكاب الخطأ، أو أن تكون غير مستعد تمامًا، أو لا تفعل الأشياء على أكمل وجه؟

1- ◯ **Yes (نعم)**

2- ◯ **No (لا)**

29. Do you tend to feel crushed even by constructive criticism, seeing it as evidence of your "ineptness"? (Ineptness: Lacking or showing a lack of skill).

(هل تميل إلى الشعور بالانكسار حتى من خلل النقد الإيجابي، حيث تعتبره دليلاً على "عدم كفاءتك"؟ (عدم الكفاءة: الافتقار إلى المهارة أو إظهار نقصها

1- ◯ **Yes (نعم)**

2- ◯ **No (لا)**

30. When you do succeed, do you think "Phew, I fooled them this time, but may not be so lucky next time"? Phew: expressing a strong reaction of relief

عندما تنجح، هل تفكر "هووه ، لقد خدعتهم هذه المرة ، لكن قد لا أكون محظوظا المرة القادمة

1- ◯ **Yes (نعم)**

2- ◯ No (لا)

31. Do you believe that other people (students, colleagues, competitors) are smarter and more capable than you?

هل تعتقد أن الاشخاص (الطلاب، الزملاء، والمنافسين) أذكى وأكثر قدرة منك؟

1- ◯ **Yes (نعم)**

2- ◯ **No (لا)**

32. Do you live in fear of being found out, discovered, or unmasked?

هل تعيش في خوف من أن يتم اكتشاف حقيقتك؟

1- ◯ **Yes (نعم)**

2- ◯ **No (لا)**

Other Questions

33. Have you been treated for a psychological illness before? (هل تعالجت من مرض نفسي من قبل ؟)

1- ◯ **Yes**

2- ◯ **No**

34. Are you still undergoing treatment? (هل مازلت تخضع للعلاج ؟)

1- ◯ **Yes**

2- ◯ **No**

35. Have you ever been smoking? (هل سبق لك التدخين؟)

1- ◯ **Yes**

2- ◯ **No**

"If the answer is NO go to question 38"

36. Are you still smoking? (هل مازلت تدخن ؟)

1- ◯ **Yes**

2- ◯ **No**

37. How many cigarettes do you smoke per day? (كم سيجارة تدخن في اليوم؟)

1- ◯ **1-5**

2- ◯ **6-10**

3- ◯ **11-20**

4- ◯ **More than 20 cigarettes**

38. Have you taken any of these? (هل أخذت أيا من هذا؟)

1- ◯ **Khat (قات)**

2- ◯ **Amphetamine (الأمفيتامين)**

3- ◯ **Other (أخرى)**

4- ◯ **I do not use any of this (أنا لا اخذ أيا من هذه)**
